# Autologous dendritic cell immunotherapy (DCVAC/PCa) added to docetaxel chemotherapy in a Phase III trial (viable) in men with advanced (mCRPC) prostate cancer

**DOI:** 10.1186/2051-1426-3-S2-P164

**Published:** 2015-11-04

**Authors:** Tomasz M Beer, Nicholas Vogelzang, Jiřina Bartůňková, Kurt Miller, William Oh, Stephane Oudard, Hardev Pandha, A Oliver Sartor, Radek Špíšek, Timothy O Toole, Niels G Borgstein, Winald R Gerritsen

**Affiliations:** 1 Oregon Health & Science University, OHSU Knight Cancer Institute, Portland, OR, USA; 2 US Oncology Research, Comprehensive Cancer Centers, Las Vegas, NV, USA; 3 University Hospital Motol, Prague, Czech Republic and SOTIO a.s., Prague, Czech Republic; 4 Charité University Medicine Berlin, Berlin, Germany; 5 Division of Hematology/Medical Oncology, The Tisch Cancer Institute, Icahn School of Medicine at Mount Sinai, New York, NY, USA; 6 Georges Pompidou European Hospital, Paris, France; 7 University of Surrey, Guildford, UK; 8 Tulane Cancer Center, New Orleans, LA, USA; 9 Sotio, LLC, Boston, MA, USA; 10 Radboud University Nijmegen Medical Centre, Nijmegen, Netherlands

## Background

Prostate cancer (PCa) is the second most common cancer, and the fifth leading cause of cancer related death among men worldwide. Immunotherapy designed to induce tumor cell specific immune responses capable of destroying tumor cells has emerged as a promising treatment modality in solid malignant tumors. Clinical and preclinical trials have shown that docetaxel chemotherapy can be combined with DCVAC/PCa immunotherapy without impairing the immune response, while Kaplan-Meier analyses showed that patients had a lower risk of death compared with both MSKCC (Hazard Ratio 0.26, 95% CI: 0.13–0.51) and Halabi (Hazard Ratio 0.33, 95% CI: 0.17–0.63) predictions[1].

## Methods

VIABLE is a randomized, double-blind, placebo-controlled, parallel-group, Phase III study to evaluate, in patients with mCRPC eligible for first-line docetaxel chemotherapy, the efficacy and safety of docetaxel chemotherapy plus DCVAC/PCa (active cellular immunotherapy based on activated dendritic cells) versus docetaxel chemotherapy plus placebo. The study was initiated in May 2014 and plans to enroll almost 1200 patients at approximately 230 sites globally. Eligible patients are required to present with metastatic castrate-resistant PCa defined by both the presence and progression of the disease, maintenance of a castrate state (less than 50 ng/dl), ECOG score 0-2, and adequate hematologic, hepatic and renal functions. All patients will receive standard of care docetaxel plus prednisone, and will be randomized 2:1 to DCVAC/PCa or placebo. Patients will be stratified by region, previous therapy and ECOG status. The primary endpoint is overall survival (OS). Assuming proportional hazards, a two-tailed level of significance of 0.05 and 80% power will be applied. Additionally this design assumes an exponential survival distribution to detect a HR = 0.792 in favor of the DCVAC/PCA group. Registration number NCT02111577, EudraCT number 2012-002814-38.

## Trial registration

ClinicalTrials.gov identifier NCT02111577. EudraCT number 2012-002814-38.
